# Supplementary material for: Efficient direct repairing of lithium- and manganese-rich cathodes by concentrated solar radiation
Source: Nat Commun. 2024 Feb 23;15:1634. doi: 10.1038/s41467-024-45754-6 (PMC10891061; doi:10.1038/s41467-024-45754-6)
Supplement: Supplementary file 1 — Supplementary Information [file 41467_2024_45754_MOESM1_ESM.pdf]

Supplementary Information for

Efficient Direct Repairing of Lithium- and manganese-rich Cathodes by Concentrated Solar Radiation

Hailong Wang[1,2], Xin Geng[1], Linyu Hu[2], Jun Wang[3], Yunkai Xu[4], Yudong Zhu[5], Zhimeng Liu[1], Jun Lu[4*], Yuanjing Lin[2*], Xin He[1,6,7*]

[1,] School of Chemical Engineering, Sichuan University, Chengdu, 610065, China
[2,] School of Microelectronics, Southern University of Science and Technology, Shenzhen 518055, China
[3,.]School of Innovation and Entrepreneurship, Southern University of Science and Technology, Shenzhen, 518055, China
[4,] College of Chemical and Biological Engineering, Zhejiang University, Hangzhou 310027, China
[5,] Department of Materials Science and Engineering, Shenzhen Key Laboratory of Full Spectral Solar Electricity Generation (FSSEG), Southern University of Science and Technology, No. 1088, Xueyuan Rd, Shenzhen, Guangdong, 518055 China
[6,] College of Electrical Engineering, Sichuan University, Chengdu, 610065, China
[7,] College of Civil Aviation Safety Engineering, Civil Aviation Flight University of China, Guanghan, 618307, China

[*] Corresponding authors:
E-mail address: Jun Lu (junzoelu@zju.edu.cn), Yuanjing Lin (linyj2020@sustech.edu.cn) and Xin He (xinhe@scu.edu.cn).

This file includes:

Supplementary Fig. 1 to 18 on pages 2 to 19;
Supplementary Table 1 to 2 on pages 20 to 21;
Supplementary Notes 1 to 3 on pages 22 to 24;
Supplementary References on page 25

Supplementary Figures

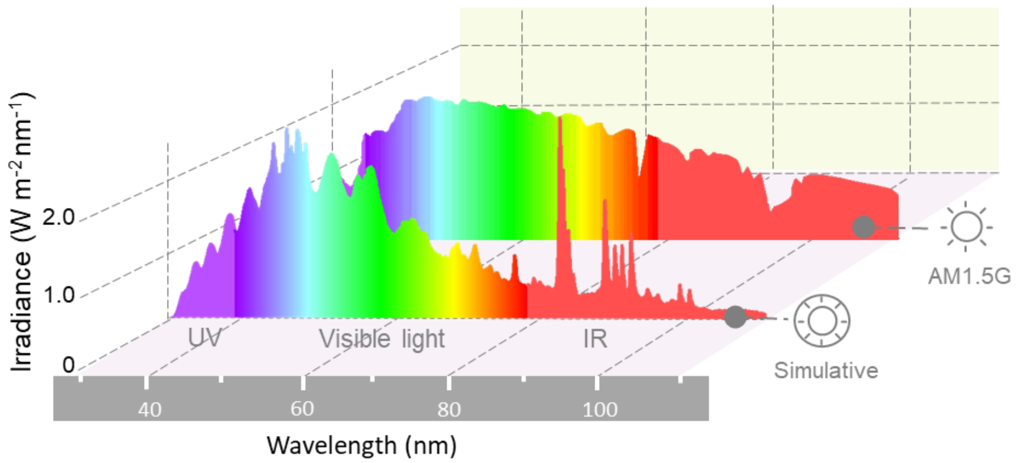

Supplementary Fig. 1 Comparison of simulated spectra with standard solar spectra (AM1.5G). The simulative spectrum is the spectral signal generated by the solar simulation equipment used in this experiment. AM1.5G is a reference spectrum widely used for standard testing of solar energy conversion systems from ASTM G173 - 03(2012) Standard Tables for Reference Solar Spectral Irradiances: Direct Normal and Hemispherical on 37°Tilted Surface[1].

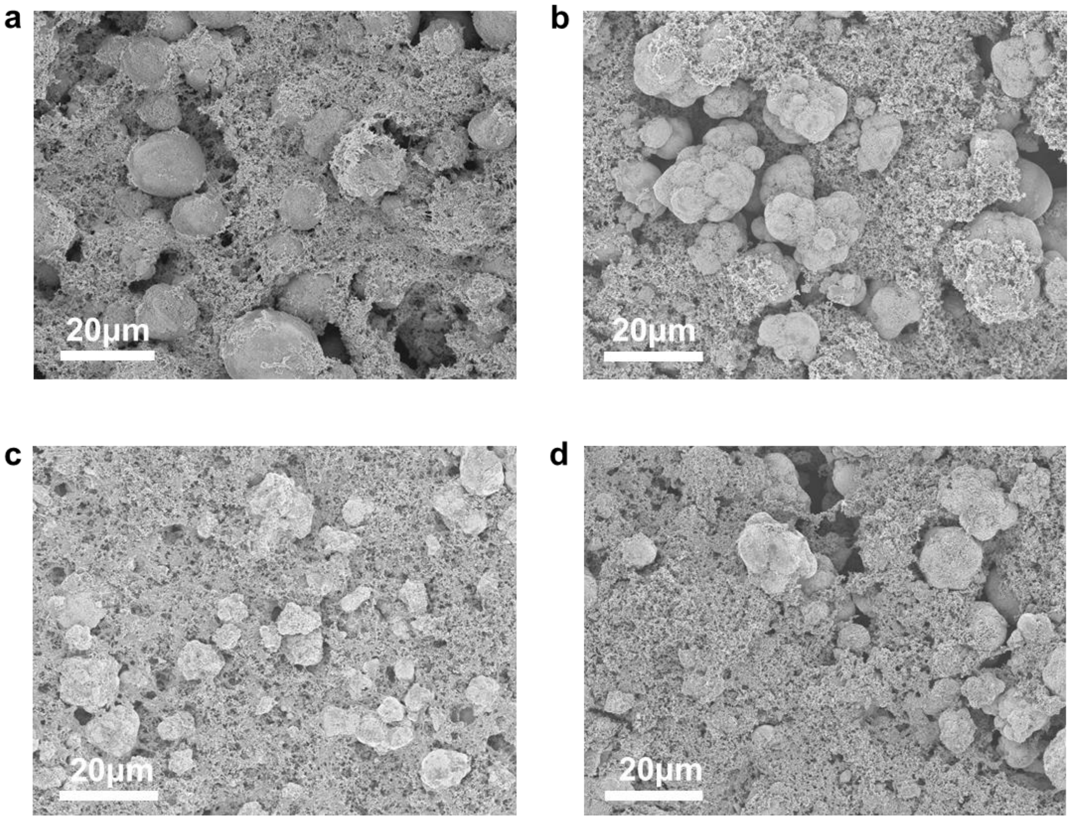

Supplementary Fig. 2 SEM images of LMRO (**a**), LMRO-S (LMRO after CSR treatment) (**b**), LMRO-50C (**c**) and LMRO-50CS (**d**) electrodes.

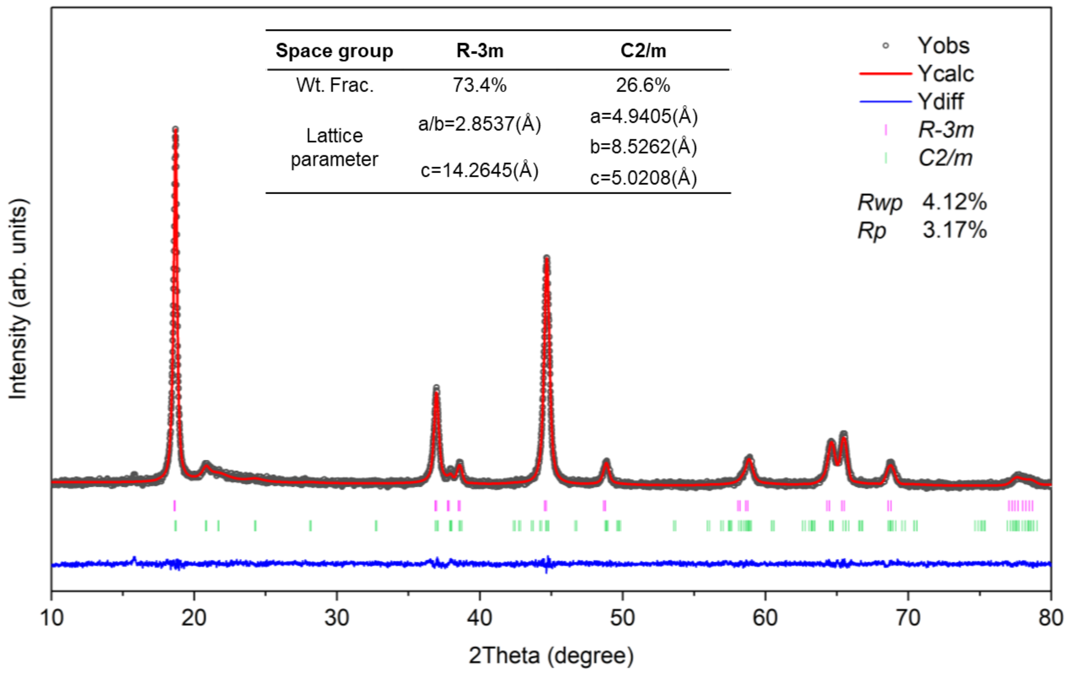

Supplementary Fig. 3 Refined X-ray powder diffraction pattern of LMRO.

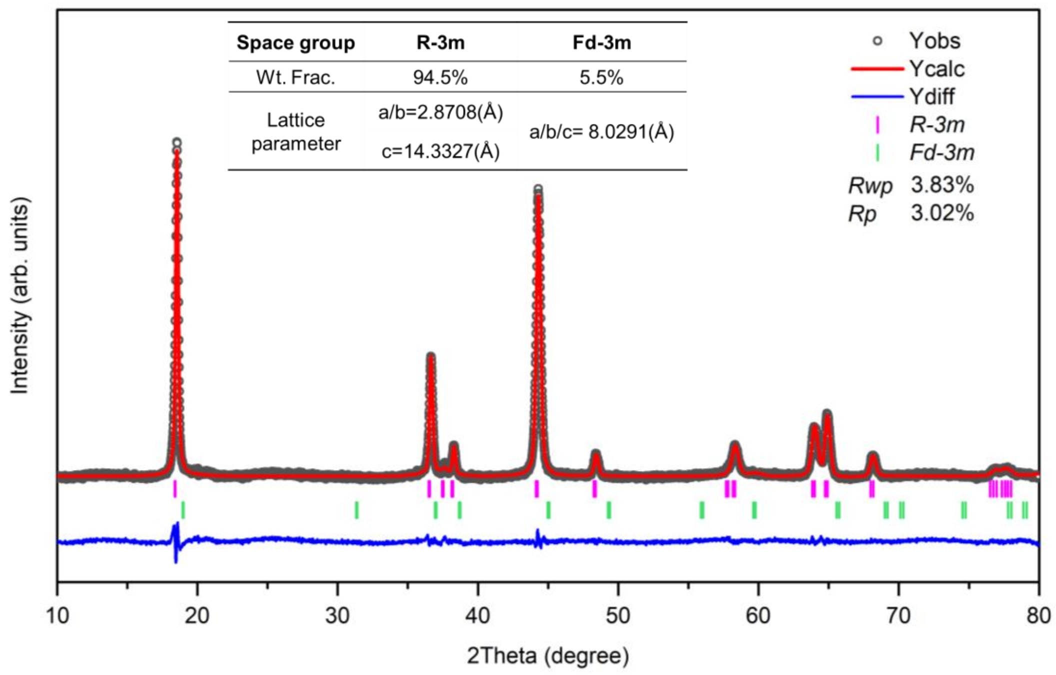

Supplementary Fig. 4 Refined X-ray powder diffraction pattern of LMRO-50C.

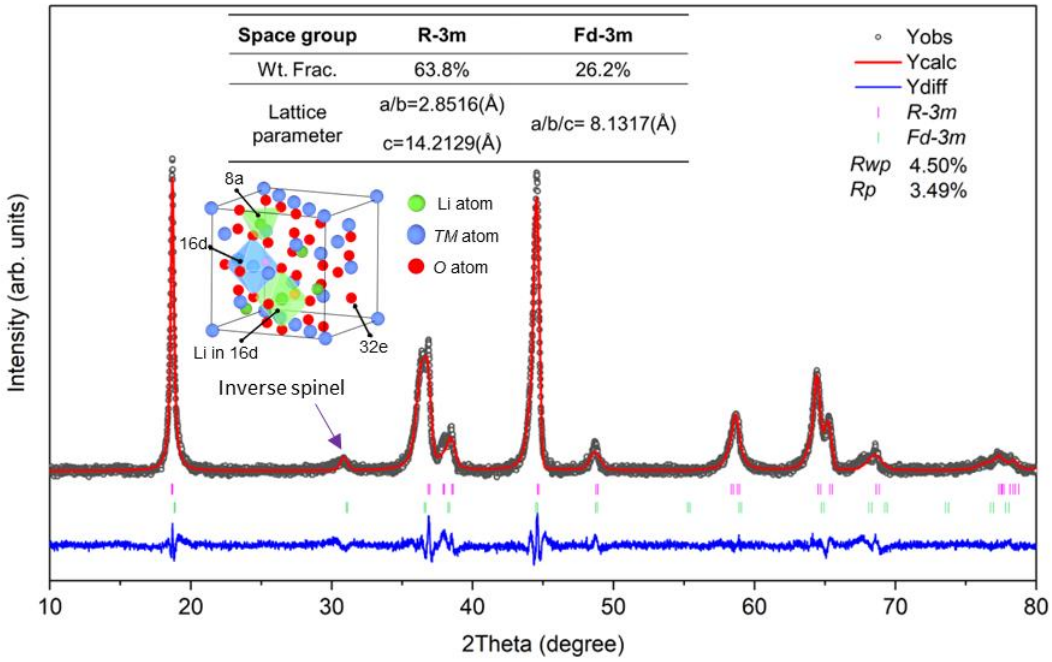

Supplementary Fig. 5 Refined X-ray powder diffraction pattern of LMRO-50CS and the illustration of the inverse spinel crystal. The blue octahedron represents the TMO6 octahedron where TMs at the 16d site; the green octahedron represents the LiO_6 octahedron where Li in the 16d site; the green tetrahedron represents LiO_4 tetrahedron where Li in 8a site.

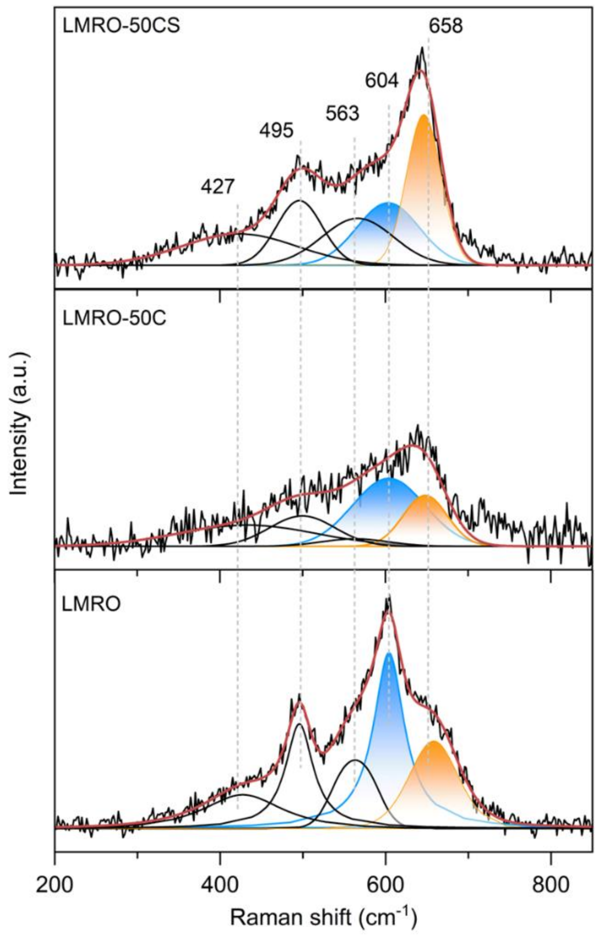

Supplementary Fig. 6 Raman spectra of LMRO, LMRO-50C and LMRO-50CS

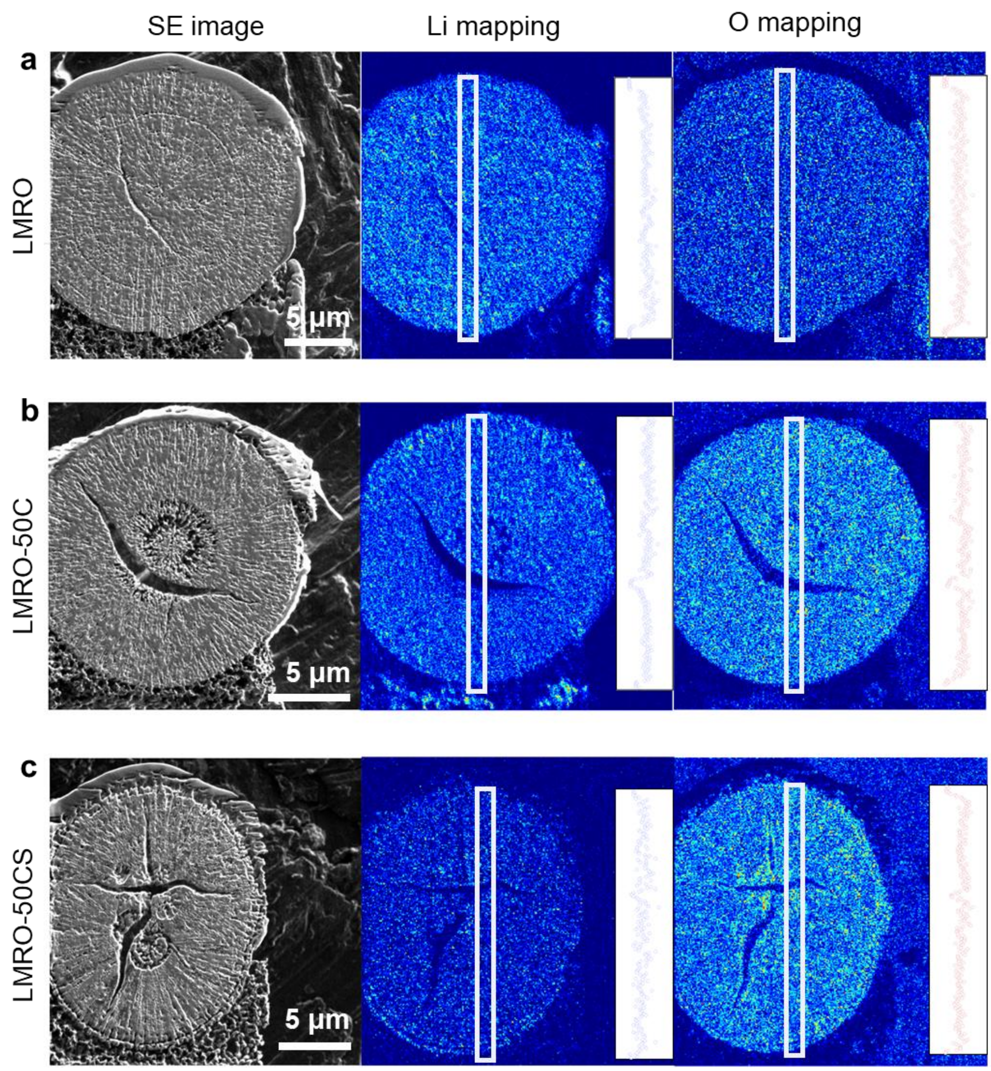

Supplementary Fig. 7 Time-of-Flight Secondary Ion Mass Spectrometry (TOF-SIMS). SE image, Li-mapping and O-mapping of LMRO (**a**), LMRO-50C (**b**), LMRO-50CS (**c**). The inset in the figure is the relative distribution of the Li and O content in the radial direction. This value is obtained by integrating the element content of the boxed area.

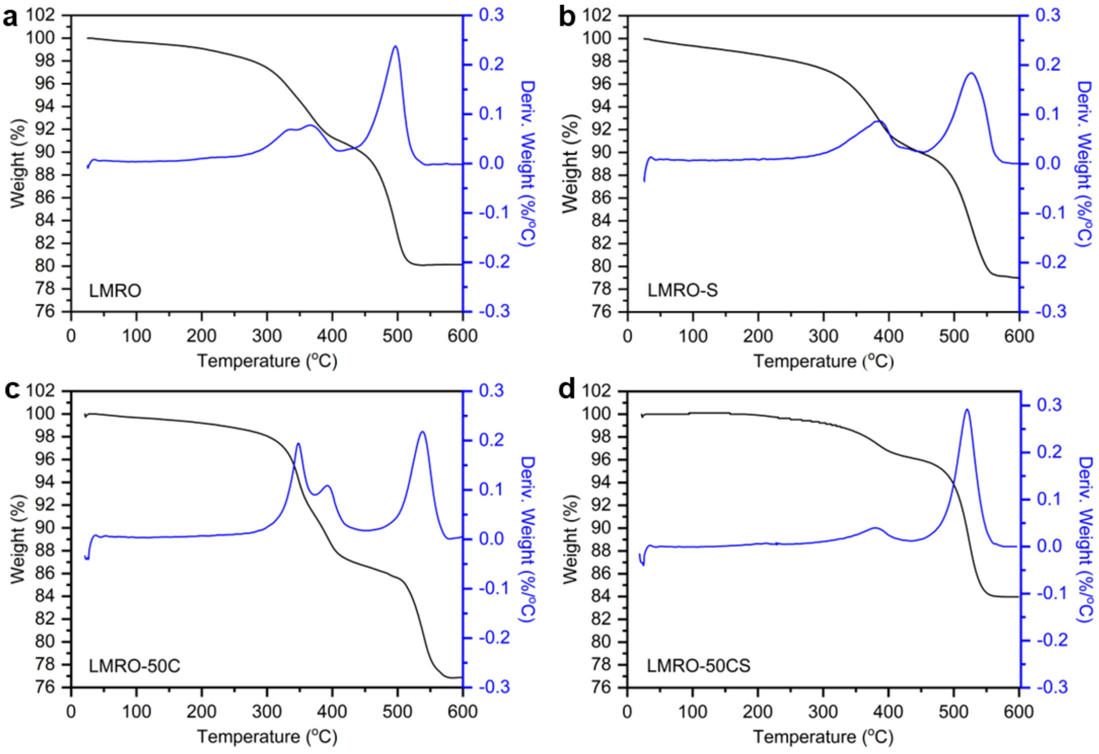

Supplementary Fig. 8 Thermogravimetric and derivative of the thermogravimetric (TG-DTG) curves of LMRO (**a**), LMRO-S (LMRO after CSR treatment) (**b**), LMRO-50C (**c**), LMRO-50CS(**d**). Temperature rise rate of 10 degrees per minute.

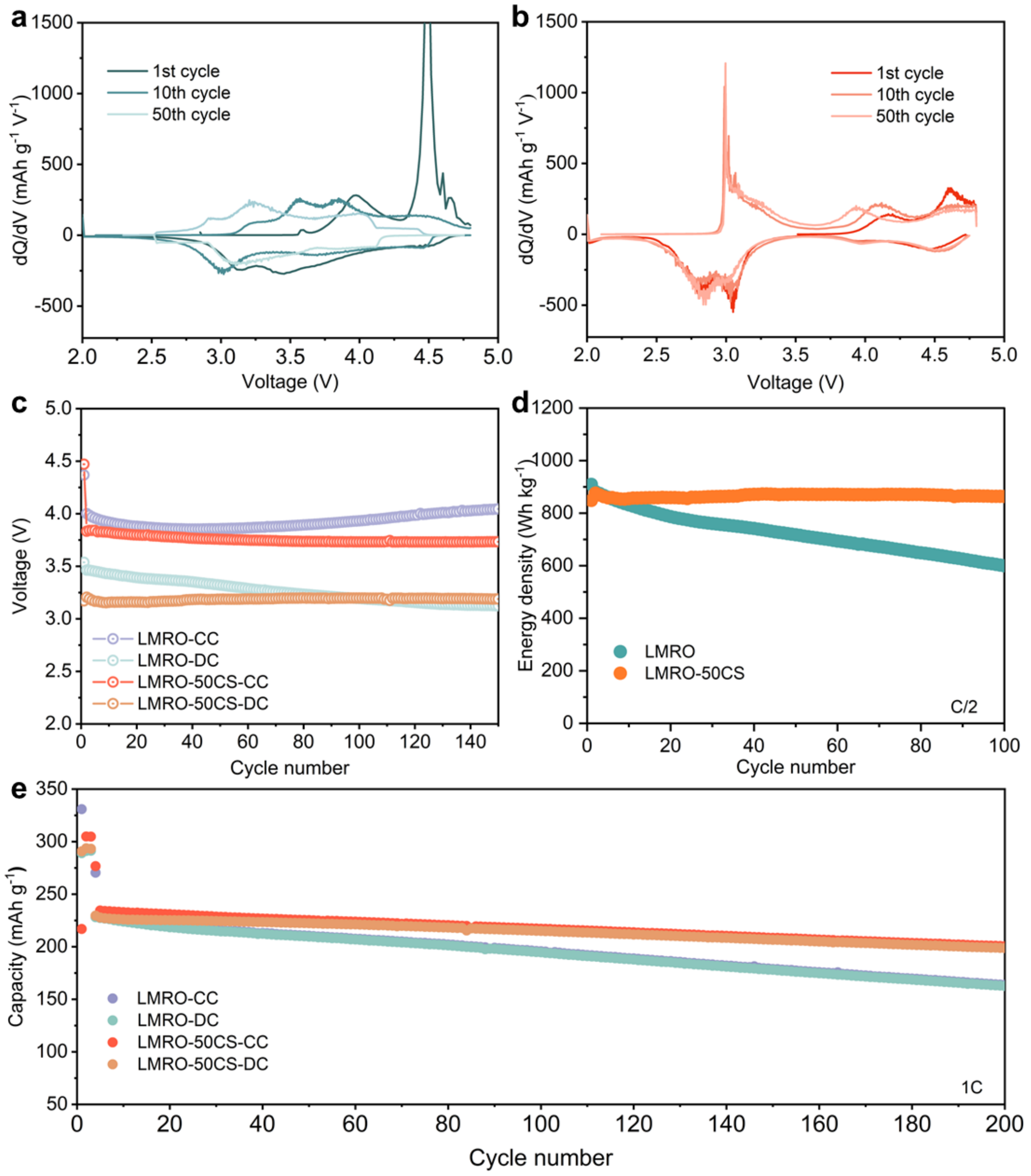

Supplementary Fig. 9 Electrochemical performance of LMRO and LMRO-50CS. The dQ/dV curves of LMRO (**a**) and LMRO-50CS (**b**) at C/10 in the first cycle and C/2 in the 10th and 50th cycle. Average voltage(**c**) and energy density (**d**) of LMRO and LMRO-50CS. **e** Cycle performance at 1C. 1C = 250mAh g^{-1}. Energy density is calculated by multiplying the discharge specific capacity with the average discharge voltage.

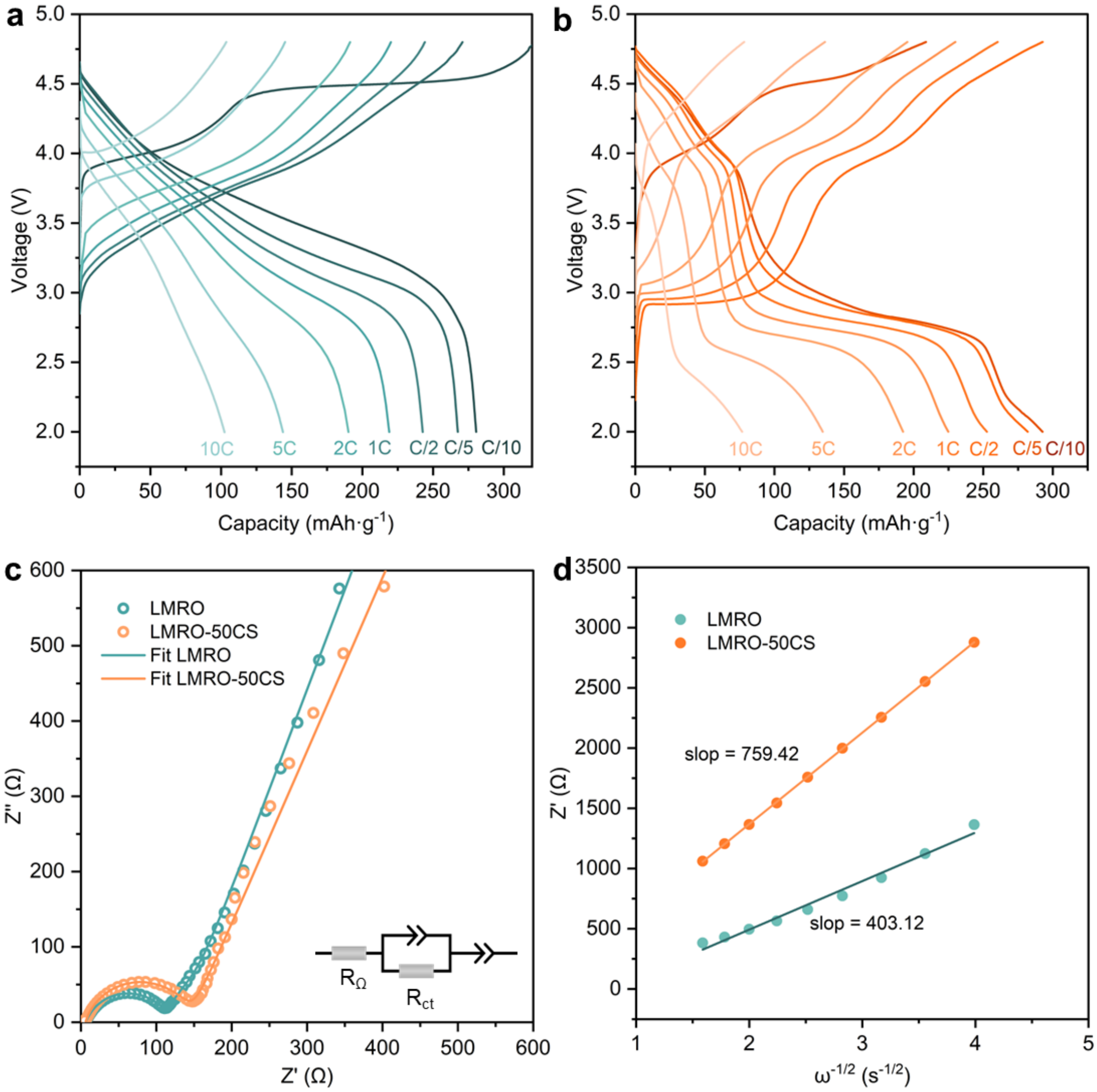

Supplementary Fig. 10 Galvanostatic charge–discharge profiles of LMRO (**a**) and LMRO-50CS (**b**) at C rates of C/10, C/5, C/2, 1C, 2C, 5C, and 10C, respectively (1C = 250 mAh g^{-1}). Nyquist plots (**c**) and profiles of Z' *vs.* $\omega^{-1/2}$ (**d**) of the LMRO and LMRO-50CS before cycling.

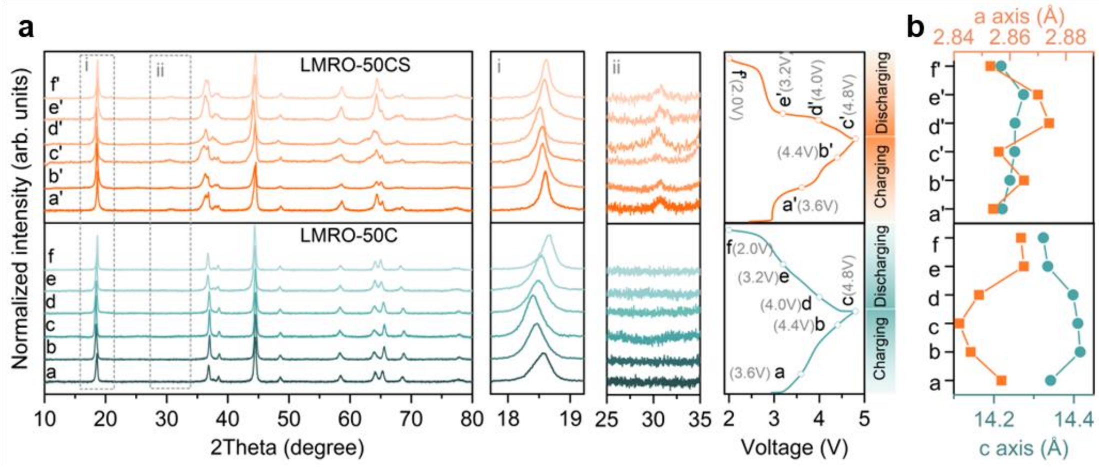

Supplementary Fig. 11 **a** Ex-situ X-ray powder diffraction (XRD) of LMRO-50C and LMRO-50CS samples at representative voltage with the enlarged view in the range of 2Theta = 17.5-19.5° (i) and 25-35° (ii), and the corresponding selected voltage stages in the second cycle profiles. **b** The corresponding lattice parameters a and c at different electrochemical states.

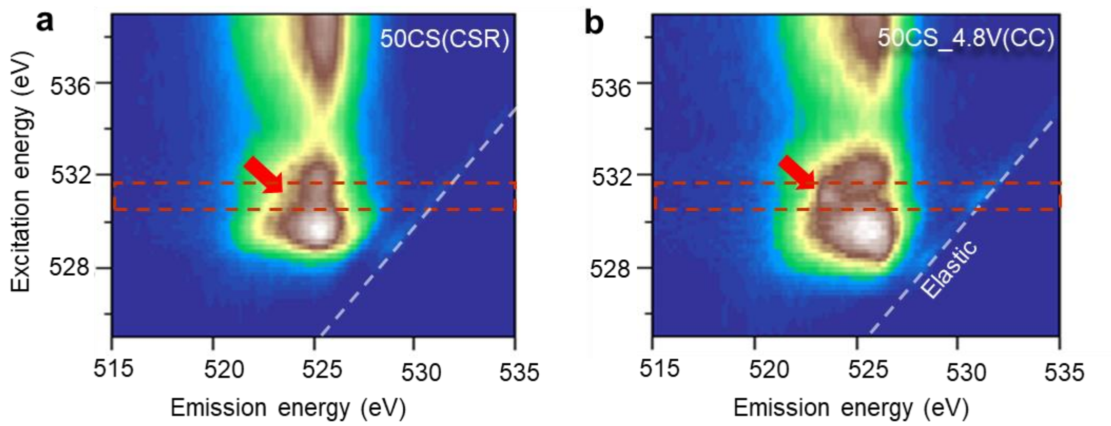

Supplementary Fig. 12 The O *K*-edge mRIXS map of LMRO-50CS (**a**) and LMRO-50CS charge to 4.8 V (**b**). The red dotted box is the selected region within the excitation energy of 530.5-531.5 eV, and the red arrow marks the characteristics of oxygen oxidation that emerges at around 531 eV excitation and 523.7 eV emission energy.

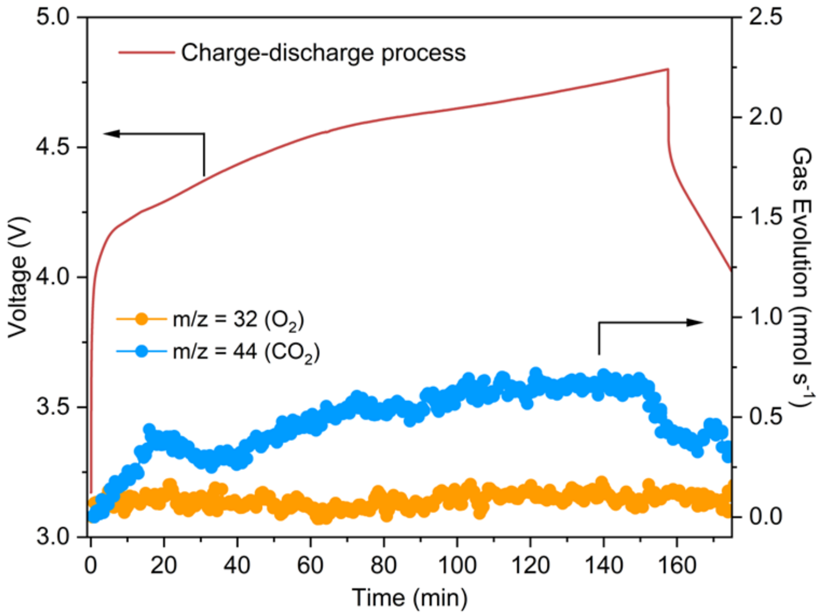

Supplementary Fig. 13 Operando DEMS profile of LMRO-50CS performed during the first charging process to 4.8 V (*vs.* Li^+/Li) at C/5.

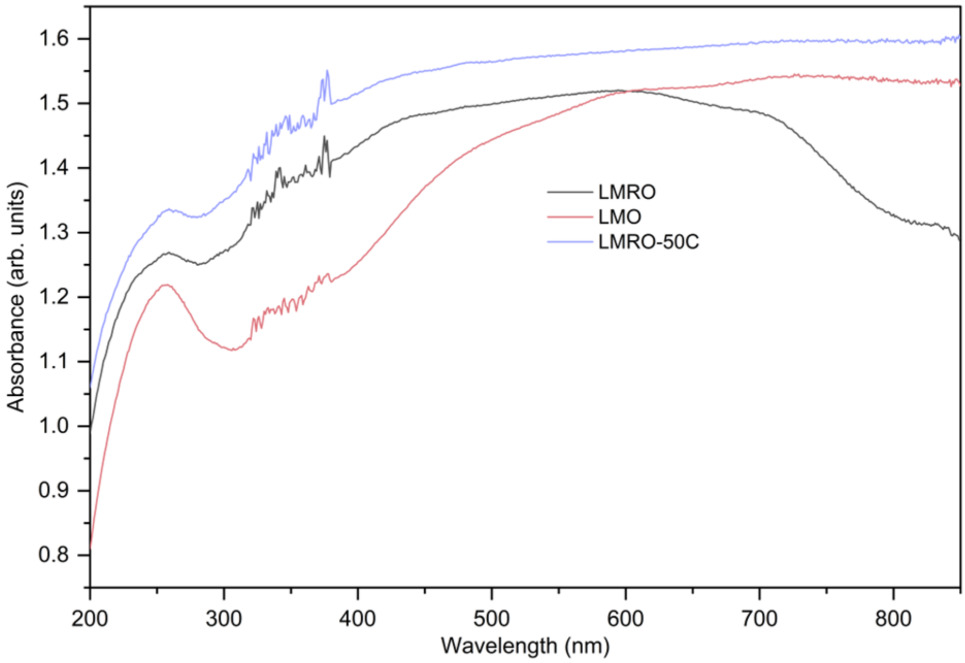

Supplementary Fig. 14 UV-vis-NIR diffuse reflectance spectra (DRS) of LMRO, LMO (spinel $LiMn_2O_4$) and LMRO-50C.

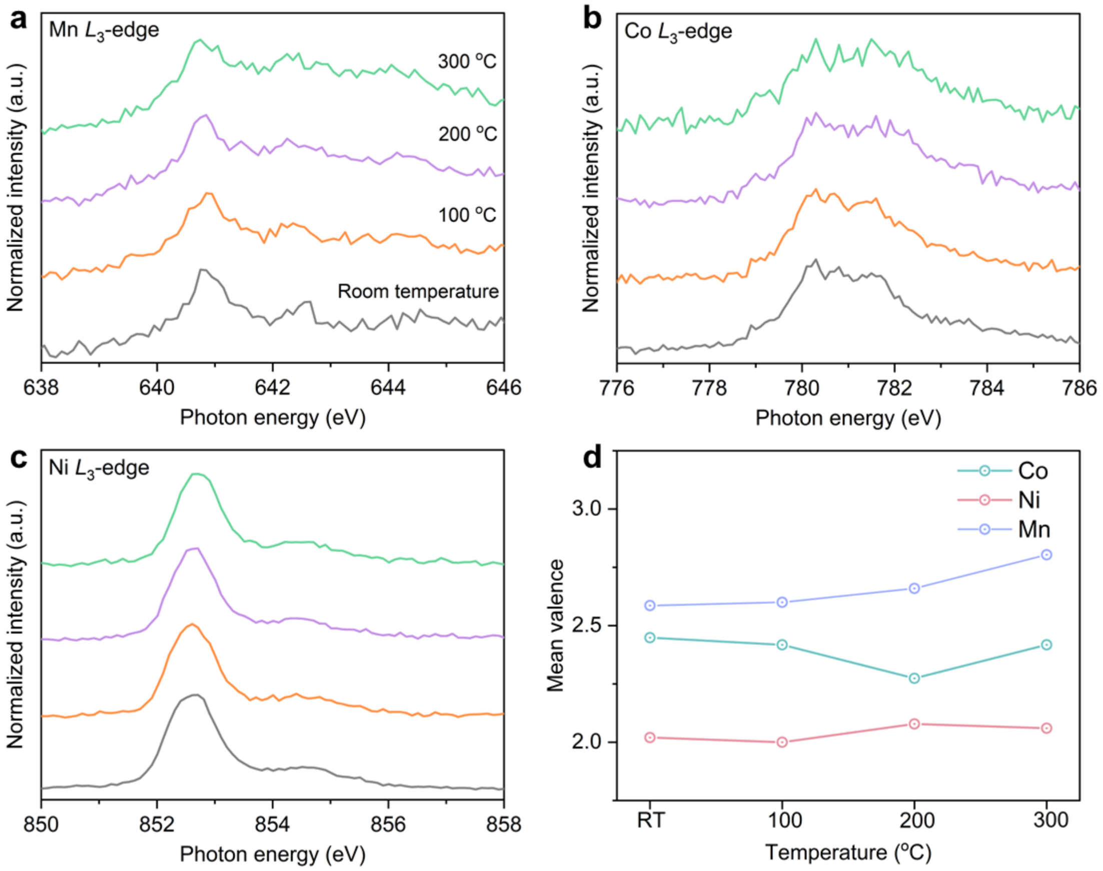

Supplementary Fig. 15 Temperature-based in-situ soft-XAS of Mn L_3-edge (**a**), Co L_3-edge (**b**), and Ni L_3-edge (**c**). **d** The quantified mean oxidation states of each *TM*, based on the fitting results of the valence distributions in the spectra.

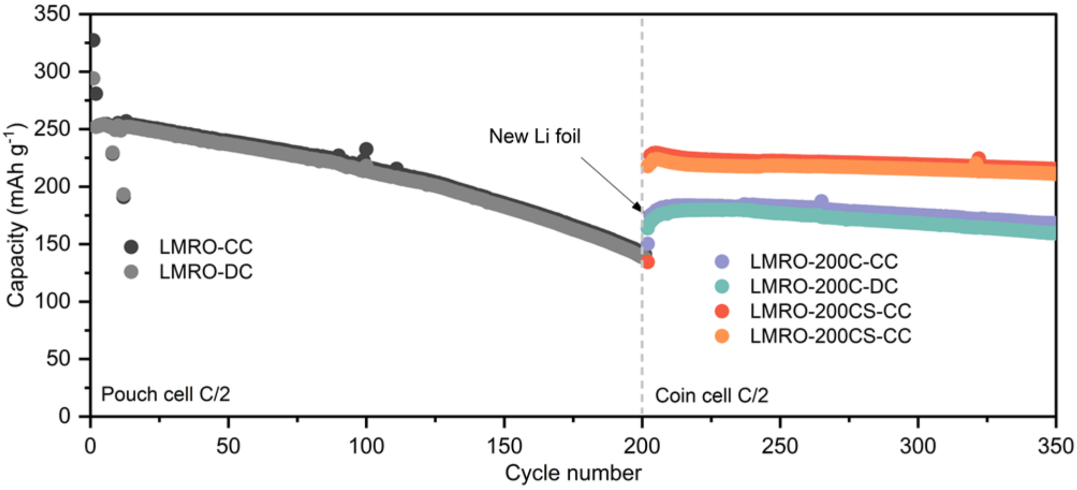

Supplementary Fig. 16 Cycle performance of LMRO, LMRO-200C and LMRO-200CS.

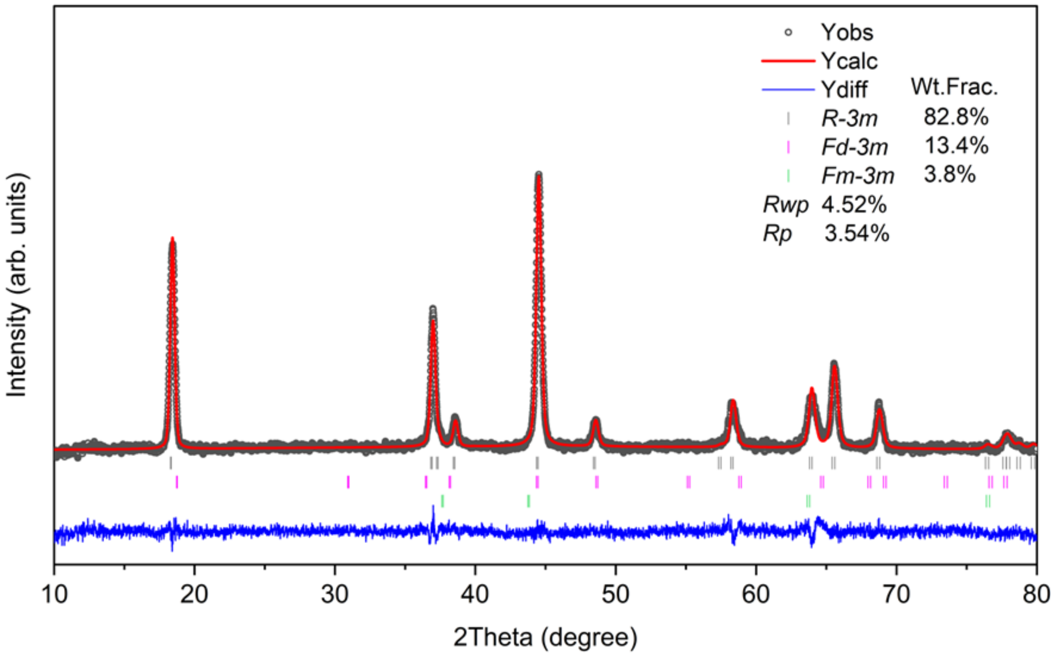

Supplementary Fig. 17 Refined X-ray powder diffraction pattern of LMRO-200C.

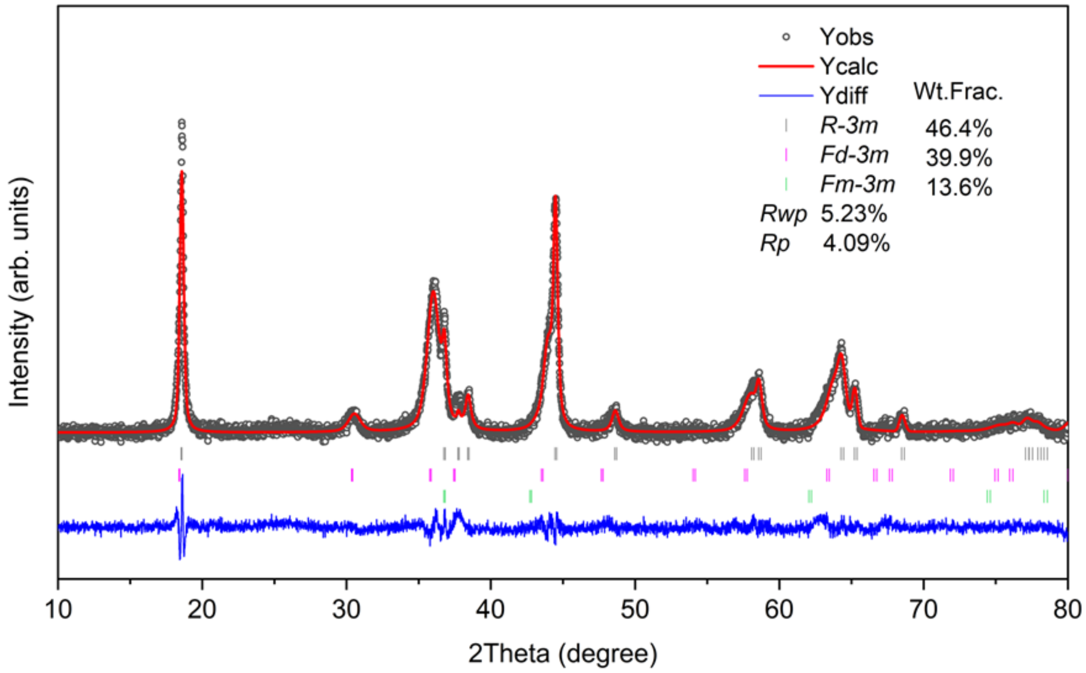

Supplementary Fig. 18 Refined X-ray powder diffraction pattern of LMRO-200CS.

Supplementary Tables

Supplementary Table 1. ICP-OES results of different LMRO cathode materials

Sample	Li	Mn	Co	Ni
LMRO	1.220	0.56	0.13	0.15
LMRO-50C	1.014	0.56	0.13	0.15
LMRO-50CS	0.762	0.56	0.11	0.13

Supplementary Table 2. Refined values of the lattice parameters

Samples		a/b (Å)	c (Å)	V (Å^3)	Rwp	Rp
LMRO-50C	a (CC to 3.6V)	2.856656± 0.000062	14.342482± 0.000506	101.361± 0.005	4.73	3.6
	b (CC to 4.4V)	2.846077± 0.000108	14.416471± 0.000841	101.131± 0.011	3.46	2.74
	c (CC to 4.8V)	2.842450± 0.000076	14.410069± 0.000713	100.828± 0.007	5.61	4.47
	d (DC to 4.0V)	2.843573± 0.000091	14.398351± 0.000937	100.826± 0.009	4.36	3.48
	e (DC to 3.2V)	2.864868± 0.000067	14.334563± 0.000494	101.888± 0.005	4.55	3.52
	f (DC to 2.0V)	2.86392± 0.000050	14.323689± 0.000412	102.133± 0.004	4.59	3.62
LMRO-50CS	a’ (CC to 3.6V)	2.853625± 0.000092	14.220580± 0.000571	100.286± 0.014	4.94	3.64
	b’ (CC to 4.4V)	2.865316± 0.000124	14.239523± 0.000551	101.244± 0.008	5.54	4.37
	c’ (CC to 4.8V)	2.855927± 0.000175	14.252447± 0.001397	100.673± 0.014	4.96	3.94
	d’ (DC to 4.0V)	2.873508± 0.000153	14.253320± 0.000956	102.988± 0.026	4.95	3.65
	e’ (DC to 3.2V)	2.870348± 0.000181	14.273851± 0.000873	101.845± 0.012	4.65	3.69
	f’ (DC to 2.0V)	2.853359± 0.000194	14.218466± 0.001170	100.253± 0.017	4.29	3.37

Supplementary Notes

Supplementary Note 1.

Transition metal mean valence analysis

Firstly, all the sXAS spectra were normalized by the height between the highest peak and the background line. Then, the quantified mean oxidation states of each *TM*, are based on the fitting results of the valence distributions in the spectra. The reference spectra of Mn[2], Ni[3] and Co[3,4] with different valence states are plotted at the bottom of Fig. 4a-c, respectively. The mean oxidation states of Mn and Ni can be precisely calculated and traced through a simple linear combination of the reference spectra. The mean valence state of cobalt is determined by analyzing the relative energy position of the experimental peaks and the reference peaks[5]

Supplementary Note 2.

The calculation of Li-ion diffusion coefficient (D_{Li+}) based on Electrochemical impedance spectroscopy (EIS)

Electrochemical impedance spectroscopy (EIS) serves as a useful supplement for investigating the kinetic processes of Li-ion. In cases where Warburg impedance is observed in the system, EIS becomes instrumental in calculating the Li-ion diffusion coefficient ($D_{Li}{}^{+}$). The diffusion coefficient of Li^+ ($D_{Li}{}^{+}$) may be estimated from the graphs in the low-frequency range, which represents the diffusion ability of Li-ion in the bulk of cathode material. The $D_{Li}{}^{+}$ values by EIS are estimated according to the below formulas:[6,7]

$$Z' = R_{\Omega} + R_{ct} + \sigma_w \omega^{-1/2} \quad (1)$$

$$D_{Li^+} = \frac{R^2T^2}{2A^2n^4C^2\sigma^2F^4} \quad (2)$$

(R) represents the ideal gas constant, (T) the temperature in kelvin, (n) the number of electrons transferred during the reaction, and (A) the area of the electrode. (F) means the Faraday constant. (C) represents the volume concentration of Li-ion, which can be simply derived from n/Vm, where Vm represents the molar volume of the substance and n represents the molar number of Li-ion. The value of σ_w is the Warburg factor, which can be obtained from the linear fit between Z' and $\omega^{-1/2}$.

Supplementary Note 3.

Calculation of optical properties of electrode

The bandgap of the three electrodes is calculated by the Tuac plot from the UV-Vis-NIR DRS spectrum. First, the relationship between the absorbance and the wavelength of the pole piece was measured by UV-VIS-NIR DRS, then the spectrogram data were smoothed using the loess method. The bandgap of the material is calculated by the Tauc method[8]. The Tauc method is based on the assumption that the energy-dependent absorbance can be expressed by the following equation:

$$(Ah\nu)^{1/n} = B(h\nu - E_g) \qquad (1)$$

Where A is the absorbance, h is the Planck constant, ν is the photon's frequency, E_g is the band gap energy, and B is a constant. The n factor depends on the nature of the electron transition and is equal to 1/2 or 2 for the direct and indirect transition band gaps, respectively. All samples in this experiment are calculated based on the direct band gap, and n is taken as 1/2. Finally, extrapolate the straight line part in the obtained graph to the abscissa axis, and the intersection point is the bandgap value[9,10]. In this experiment, the E_g of LMRO, LMRO-50C and LMO (spinel $LiMn_2O_4$) were 1.112 eV, 1.163 eV and 0.836 eV, respectively.

The flat-band potential (E_f) of the electrode is determined by the Mott-Schottky equation. The Mott-Schottky equation describes the relationship between the space charge layer differential capacitance C_{SC} of a semiconductor and the semiconductor surface potential E_f, which can be expressed as follows:

For n-type semiconductor: $$C_{sc}^{-2} = \frac{2}{\varepsilon\varepsilon_0 e N_D}\left(E - E_f - \frac{kT}{e}\right) \qquad (2)$$

For p-type semiconductor: $$C_{sc}^{-2} = -\frac{2}{\varepsilon\varepsilon_0 e N_A}\left(E - E_f - \frac{kT}{e}\right) \qquad (3)$$

Where the E_f is flat-band potential, N_D and N_A are carrier concentrations, ε is the relative dielectric constant, ε is the vacuum dielectric constant (8.85×10^{-12} F m^{-1}), k is Boltzmann constant (1.380649×10^{-23} J K^{-1}), T is the absolute temperature and e is the unit charge (1.6×10^{-19} C). In the C_{SC}^{-2}-E diagram, the value of the potential at the intersection of the straight-line extension and the horizontal coordinate is the flat-band potential. In this experiment, the materials measured are all n-type semiconductors with E_f of -1.065 V, -0.792 V, and -0.924 V for LMRO, LMO-50C and LMO. According to the property of n-type semiconductor that the E_{CB} of is lower (0.1 eV) than its E_f, the conduction band potential (E_{CB}) can be estimated as -1.165 V, -0.892 V and -1.024 V *vs* Ag/AgCl

for LMRO, LMRO-50C and LMO, respectively. Then the valence band potential (E_{VB}) can be calculated by the following equation:

$$E_{CB} - E_{VB} = E_g \quad (4)$$

Therefore, the E_{VB} of the LMRO, LMRO-50C and LMO are 0.377 V, 0.705 V and 0.786 V *vs.* Ag/AgCl.

Then all voltages can be converted to a state where the Li^+/Li standard potential is the reference voltage according to the standard potential difference as shown below:

The E_f of LMRO, LMRO-50C and LMO are 2.179 V, 2.452 V and 2.320 V *vs* Li^+/Li, respectively.

The E_{CB} of LMRO, LMRO-50C and LMO are 2.079 V, 2.352 V and 2.220 V *vs* Li^+/Li, respectively.

The E_{VB} of LMRO, LMRO-50C and LMO are 3.191 V, 3.515 V and 3.056 V *vs.* Li^+/Li, respectively.

Supplementary References

1. Mambrini, T. *et al.* Photovoltaic yield: Correction method for the mismatch between the solar spectrum and the reference ASTMG AM1.5G spectrum. *EPJ Photovoltaics* **6**, 60701 (2015).
2. Dai, K. *et al.* High Reversibility of Lattice Oxygen Redox Quantified by Direct Bulk Probes of Both Anionic and Cationic Redox Reactions. *Joule* **3**, 518–541 (2019).
3. Lee, G. *et al.* Reversible Anionic Redox Activities in Conventional $LiNi_{1/3}$ $Co_{1/3}$ $Mn_{1/3}$ O_2 Cathodes. *Angew. Chemie* **132**, 8759–8766 (2020).
4. Huang, Y. C. *et al.* In Situ/ Operando Soft X-ray Spectroscopic Identification of a Co4+Intermediate in the Oxygen Evolution Reaction of Defective Co3O4Nanosheets. *J. Phys. Chem. Lett.* **13**, 8386–8396 (2022).
5. Li, Q. *et al.* Quantitative probe of the transition metal redox in battery electrodes through soft x-ray absorption spectroscopy. *J. Phys. D. Appl. Phys.* **49**, 413003 (2016).
6. Ho, C., Raistrick, I. D. & Huggins, R. A. Application of A-C Techniques to the Study of Lithium Diffusion in Tungsten Trioxide Thin Films. *J. Electrochem. Soc.* **127**, 343–350 (1980).
7. Jafta, C. J., Ozoemena, K. I., Mathe, M. K. & Roos, W. D. Synthesis, characterisation and electrochemical intercalation kinetics of nanostructured aluminium-doped $Li[Li_{0.2}Mn_{0.54}Ni_{0.13}Co_{0.13}]O_2$ cathode material for lithium ion battery. *Electrochim. Acta* **85**, 411–422 (2012).
8. Paulraj, V., Swami, B. & Kamala Bharathi, K. Growth behavior, work function, and band gap tuning of nanocrystalline $LiMn_2O_4$ thin films. *Appl. Phys. Lett.* **115**, 093901 (2019).
9. Makuła, P., Pacia, M. & Macyk, W. How To Correctly Determine the Band Gap Energy of Modified Semiconductor Photocatalysts Based on UV-Vis Spectra. *J. Phys. Chem. Lett.* **9**, 6814–6817 (2018).
10. Yuan, T. *et al.* Mild and metal-free Birch-type hydrogenation of (hetero)arenes with boron carbonitride in water. *Nat. Catal.* **5**, 1157–1168 (2022).
